# Controlling Polymer Electrolyte Interfacial Morphology through Chemical Interactions

**DOI:** 10.1021/acs.chemmater.5c01738

**Published:** 2025-08-18

**Authors:** Joseph A. Dura, Sangcheol Kim, Kirt A. Page, Christopher L. Soles

**Affiliations:** † NIST Center for Neutron Research, 100 Bureau Dr. Gaithersburg, Maryland 20899, United States; ‡ Wiss, Janney, Elstner Associates, Inc. 330 Pfingsten Road, Northbrook, Illinois 60062, United States; § Materials and Manufacturing Directorate, Air Force Research Laboratory, 5135 Pearson Road, Building 10, Wright-Patterson AFB, Dayton, Ohio 45433, United States; ∥ Cornell University, Cornell High Energy Synchrotron Source, 161 Synchrotron Drive, Ithaca, New York US 14853-0001, United States; ⊥ NIST Materials Measurement Laboratory, 100 Bureau Dr., Gaithersburg, Maryland 20899, United States; ▲ Av Inc., 4401 Dayton-Xenia Rd.. Dayton, Ohio 45432, United States

## Abstract

Owing to its unique mechanical properties, chemical resistance, and ion conductivity, Nafion is one of the most widely used polymer electrolytes. In hydrogen fuel cells, it constitutes both the macroscopic membrane separating the anode and the cathode, and as a thin film, Nafion appears as a binder in the catalyst layer where conductive ionic pathways must intimately interface with platinum catalyst particles, electrically conductive carbon particles, and porous surfaces that facilitate the transport of gases. Residing at the intersection of this diverse range of materials, the ionomer’s interfacial structure influences interfacial impedance and thus device performance. This interface structure has been widely investigated on model surfaces with neutron reflectometry and other techniques, resulting in the discovery of a multilamellar structure at the interface with hydrophilic materials, or a single water-rich layer at the interface with, e.g., metals, favoring tangential vs perpendicular ion transport, respectively. Here we demonstrate that self-assembled monolayers, SAMs, which can coat various surfaces, can control whether single or multiple lamellae occur. These interfacial structures can be further modified through acid–base interactions by protonating the terminal amine group of a SAM at low pH. This establishes a methodology to control the interfacial ionic transport pathways in Nafion and determine the interfacial impedance.

## Graphical abstract

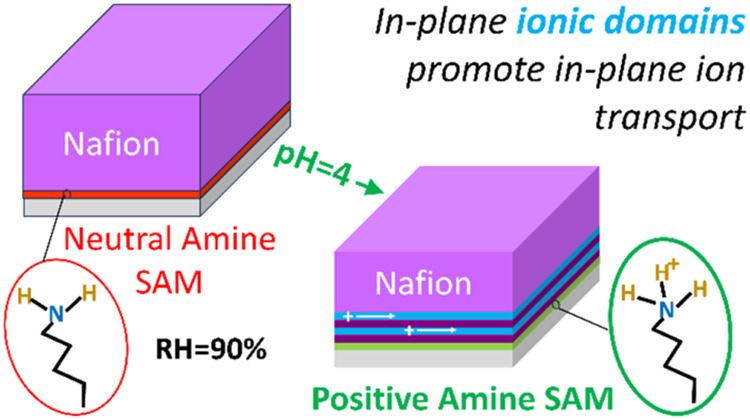

## Introduction

Nafion is the most widely used polymer electrolyte due to its excellent ion conductivity, imperviousness to gas crossover, chemical stability, and durable mechanical properties. Nafion has become the most common benchmark against which new ionomers are compared. These properties stem from its unique molecular structure and the resulting phase-separated nanoscale structure of the ionically conducting domains. Nafion consists of a fluorocarbon backbone decorated at random locations by a perfluoro ether side chain terminated by sulfonic acid (Figure 1). The hydrophobic backbones form nanoscale semicrystalline regions that, when hydrated, phase segregate from the hydrophilic ionic domains containing sulfonic acid and water.^1^ While determining the exact morphology in bulk is difficult, the current understanding is that it consists of bundles of water-rich cylindrical domains dispersed in a polymer matrix^2^ although alternative views are elongated polymer aggregates that bundle,^3^ possibly into ribbon-like units^4^ supported by cryo-TEM results.^5^ Regardless of the different morphological models of Nafion, its function as a fuel cell membrane stems from the ability of the H^+^ to transport through the membrane by ion hopping between sulfonic acid sites aided by motions of the side chains and by Grotthuss hopping enabled by the large amount of water in the ionic domains.^6^

**Figure 1:**
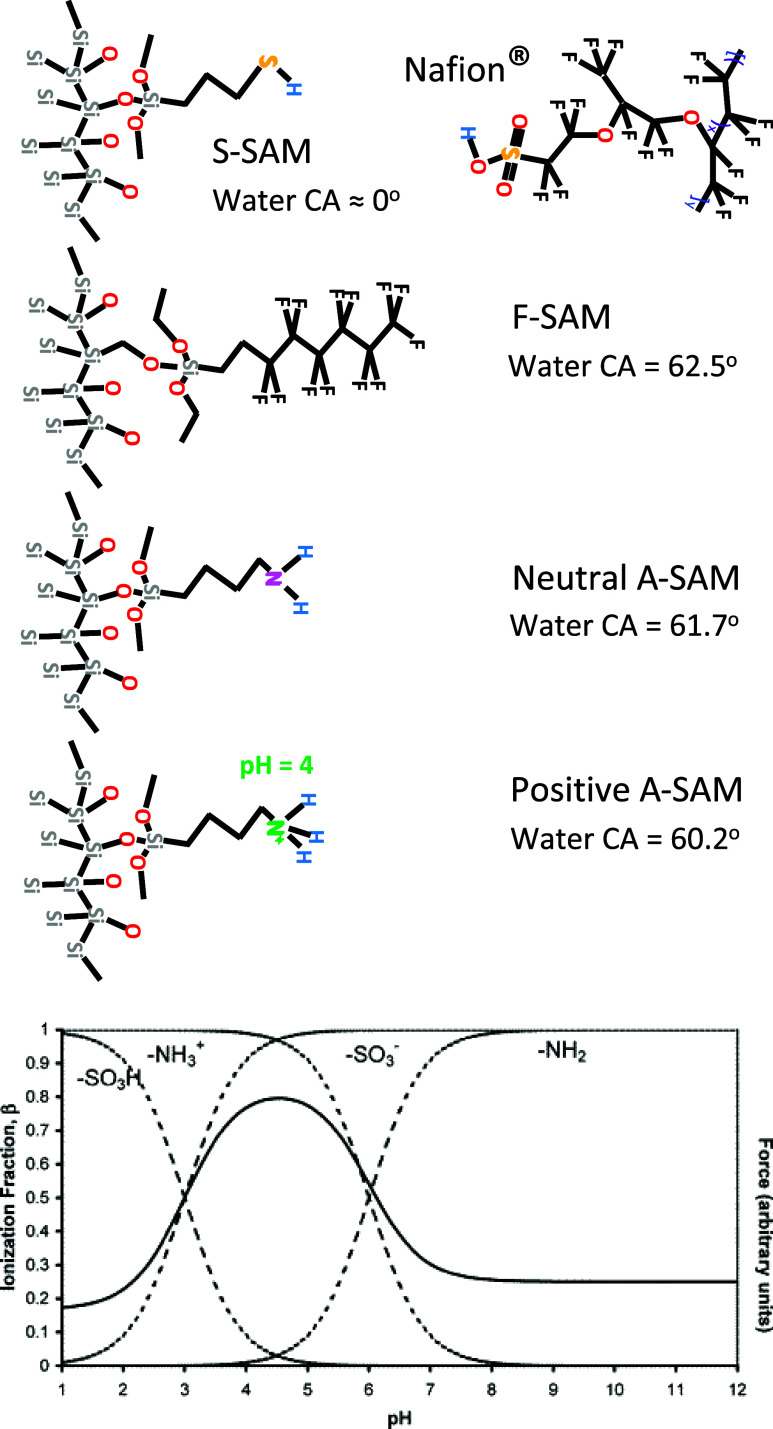
Chemical structures of Nafion and the four SAMs, attached to Si used in this study, along with their water contact angles. At the bottom is shown the ionization fraction and adhesive force between an amine-coated AFM tip and sulfonic acid-coated substrate as a function of pH. Reproduced from [38] Copyright 2005 American Chemical Society.

Nafion’s transport and water uptake properties also enable a wide range of applications that involve Nafion interfaces, primarily as a binder in the electrode catalyst layer in PEM fuel cells, where Nafion coatings as thin as a few nm are observed.^7^ It is well-known that the water uptake^8–10^ and transport properties^11^ of thin polymer films deviate from their bulk values and it is believed that this interfacial confinement in thin Nafion films is a source of interfacial impedance in the catalyst layer. The performance of a fuel cell critically depends on the efficient transport of gases, ions, and electrons through these interfaces. It is therefore important to understand and potentially control the morphology of ionic domains in the few-nanometer-thin Nafion films, which are present in the catalyst layer (where gas, ions, and electrons come together for either oxidation or reduction reactions).^7^

To achieve this understanding, it is important to determine how the different surface interactions and thin film confinement alter the interfacial structure of Nafion’s ionic domains and the corresponding water uptake. Neutron reflectometry, NR, is a diffraction-based probe that is used to determine a depth profile of composition in terms of the scattering length density, SLD, averaged in the plane of flat samples such as thin films. It is sensitive to features with thicknesses on the order of 1 to 500 nm with angstrom-level precision and is an ideal in situ technique to study buried interfaces within complex sample environments.^8,9,12–19^ NR is particularly useful for determining water content as a function of depth due to the isotopic differences in scattering between hydrogen and deuterium, which is also of use in characterizing hydrocarbons, such as many polymers. Measurements of a sample under the same parameters using H_2_O and D_2_O can determine depth profiles of both the water volume fraction and the SLD of the nonwater component, from which its composition can often be inferred.^13^

The ionic domain structure of Nafion can be altered near interfaces in different ways, which can depend on the material with which it is in contact. It was discovered that on SiO_2_, the ionic domains of hydrated Nafion reorganize to form multiple lamellae parallel to the interface, beginning with a water-rich ionic domain followed by Nafion rich layer, which repeats with decreasing compositional variations away from the interface and partially persists upon drying.^8^ These results were later confirmed in a study that samples at finer steps in humidity and with both H_2_O and D_2_O^20^ and with coarse grain modeling simulations.^21,22^ It was demonstrated that in these lamellae the Nafion phase separates, with more sulfonic acid in the water-rich layers and more fluorocarbon tails in the water-poor layers.^13^ Other hydrophilic surfaces such as organosilicate glass (OSG) coatings can also promote the multilamellar structure.^23^ However, on metals such as Au and Pt^8,24–26^ and untreated C,^27^ a single water-rich lamella exists at the interface. It was suggested that while the sulfonate groups of Nafion bind to the surface of Pt^28^ and a spring model was introduced to describe their proximity to the Pt as a function of the countercation and potential,^29^ fewer lamella form on these surfaces than on e.g., SiO_2_ due to the weaker attraction of water and sulfonate groups.^8^ Another study shows that there is not a water-rich layer at the interface with C that was treated to be hydrophobic.^30^ A slightly water-poor interface was observed on nitrogen (N)-modified carbon surfaces.^31^

The effects of thickness on water uptake in the various layers of Nafion on SiO_2_ were also studied.^9^ It was found that there are 3 thickness regimes. The lamellar regime occurs when the film is truncated at *T*_Naf_ ≤ 7 nm, to include only the multiple lamellae, which have bulklike water uptake when averaged over the film. *T*_Naf_ is the equivalent thickness of Nafion in the sample, i.e., the amount of Nafion it contains, expressed as a thickness at bulk density. In slightly thicker films, 7 nm < *T*_Naf_ < 60 nm, (the thin film regime) a thicker uniform layer of Nafion is on top of the multiple lamellae. The water uptake of both this noninterfacial layer and the lamellae increases with thickness but are both less than in bulk Nafion. For much thicker films, when *T*_Naf_ ≥ 60 nm, (the thick film regime) water uptake is bulklike in the nonlamellar portion and much higher in the lamellae.^9^ This water uptake with thickness can influence transport properties, affecting both the solubility and diffusivity of water in the Nafion film^11^ and the proton conductivity.^9,10,32,33^ Higher temperatures were also investigated. Nafion deposited on the native oxide of Si and annealed and measured at 80 °C at a variety of humidities was fit with 2 interfacial layers.^34^ Also, for 16 nm (dry) Nafion films on SiO_2_, increased water uptake and swelling were observed as the temperature is raised from 25 to 60 °C at constant *RH*∼96%. Upon cooling again to 25 °C, 73% of the added swelling is retained. This indicated a change in structure for exposure to high RH and high temperatures, with a slower dynamics upon returning to the lower temperature state.^35^ In a study of the dynamics of swelling of Nafion in liquid D_2_O, three time scales for swelling were observed with saturation times of roughly ∼1200, 4500, and 15000 s, indicating different physical mechanisms.^36^ The properties of ultrathin films can be modified by depositing them through self-assembly rather than the spin coating used here.^37^

It would be useful to control these important interfacial structures of Nafion, and thereby their related transport properties, on any device interface regardless of its composition. The goal of the current study is to illustrate that self-assembled monolayers, SAMs, which can be applied to a wide variety of materials and device interfaces, can be used to tune the surface interactions of the substrate with Nafion and thereby control the interfacial structure. We will also show that in some cases the interaction can be further tuned by changing the charge on the terminal group of the SAM, potentially enhancing the lateral control over the nanomorphology on a given surface. We will utilize two SAMs intended to mimic the two components of Nafion, i.e., the sulfonic acid group and the fluorocarbon backbone. As shown in Figure 1, (3-mercaptopropyl) trimethoxysilane (MPTMS) is a SAM that consists of a short hydrocarbon chain with silane on one end to bind to Si substrates and a thiol group on the other end, which can be readily converted to a sulfonic acid, similar to side groups of Nafion, that has similar interactions with water. We envision that such a surface treatment would attract a water-rich layer, which in turn attracts the sulfonic acid-terminated side chains of Nafion, creating an interface that would propagate the lamellar phase segregation from this interface into the film. The other SAM, (tridecafluoro-1,1,2,2-tetrahydrooctyl) triethoxysilane, creates substrates decorated with fluorocarbon chains, which is conjectured to do the reverse, i.e., to be miscible with the fluorocarbons of the Nafion backbone, leading to phase segregation and the multilamellar interfacial structure.

Finally, as a control, a neutral primary amine (−NH_2_) terminated SAM, 3-aminopropyltrimethoxysilane (APTMS), which has intermediate hydrophilicity and is not expected to exhibit strong preference for either the fluorocarbon or sulfonate moieties of Nafion, is explored. However, the interaction strength between the amine group and the sulfonate in the Nafion can be significantly enhanced by ionizing the amine at low pH, converting the NH_2_ to an NH_3_^+^ ion, which is expected to induce a strong acid–base interaction with Nafion’s sulfonate groups^38^ as seen in the inset to Figure 1, and was also investigated.

## Experimental Section

### Sample Preparation

To deposit the S-SAM layer, a Si substrate was immersed in a solution of (3-mercaptopropyl) trimethoxysilane (3-MPTMS) in toluene for 2 to 4 h. It was rinsed with toluene, acetone, and ethanol sequentially. This thiol-treated Si wafer shows a water contact angle of 56.2° after 2 h and 62.5° after 4 h. The sample was oxidized in hydrogen peroxide (30%) at 60 °C for 1 h and then rinsed with DI water, after which static water contact angle measurements, made using a Kruss G2, showed the water contact angle was 20°. It was then dipped in sulfuric acid (10 wt %) in DI water for 1 h to form the SO_3_H surface with a water contact angle near 0°.

To form the F-SAM, after the Si substrate is treated with hydrogen peroxide or ultraviolet (UV)-Ozone exposure, it is exposed to (tridecafluoro-1,1,2,2-tetrahydrooctyl) triethoxysilane (TFOTS) vapor in rough vacuum for 1 h and rinsed with toluene, acetone, and ethanol sequentially to coat the Si substrate with fluorocarbon chains. At this point, the water contact angle is 58.9 to 62.5°. It is believed that the reduced water contact angle indicates that the wafer is partially or loosely covered with F-SAM.

The amine SAM layers were formed by exposing a Si substrate to 3-aminopropyltrimethoxysilane (APTMS) in rough vacuum for 1.5 h and then rinsing with toluene, acetone, and ethanol sequentially, giving a water contact angle of 55.9°. After the sample was annealed at 110 °C for 15 min, the water contact angle increased to 61.7°. One sample was left at this stage while the other was covered in a pH = 4 buffer for 10 min, at which point the contact angle was 60.2°. Static water contact angle measurements showed that the surface coverage of these samples was sufficient to modify the surface properties compared to the otherwise native oxide-terminated Si substrate

Nafion solutions (1100 equiv molecular mass, 20% by mass dissolved in a mixture of lower aliphatic alcohols and water, containing 34% by mass water, from Sigma-Aldrich Co.) were diluted in anhydrous ethanol at a ratio of 1:16 by volume. After thorough mixing, the viscous Nafion solution was dispensed onto the SAM-modified substrates and immediately spin-cast at 367 rad/s for 40s. The films were then annealed in a vacuum oven for 1 h at 60 °C, below the α-relaxation temperature of Nafion.

### Neutron Reflectometry

NR^12^ determines a material property called the neutron scattering length density, SLD, as a function of depth, *z*, averaged in the plane of the sample over the projected coherence length of the neutron.^39^ The SLD is derived from the composition.

1 $$\mathrm{SLD}\;\left(z\right)=\sum_{j}b_{c,j}N_{j}\left(z\right)$$

Here, *b*_*c*,*j*_ is the bound coherent scattering length, and *N*_*j*_ *(z)* is the number density of isotope *j* at distance *z* from a given interface. Since the scattering lengths of the constituent materials can be determined from their composition and densities using the known *b*_*c*_, this SLD profile can typically be interpreted as a material depth profile. NR is particularly sensitive to the water content by utilizing the isotopic difference in the neutron SLD between hydrogen and deuterium. The SLD of H_2_O is −0.561 × 10^–4^ nm^–2^, whereas the SLD of D_2_O is 6.402 × 10^–4^ nm^–2^, and the SLD of dry 1100 eq. wt. Nafion is 4.158 × 10^–4^ nm^–2^.

In NR, one measures the reflected neutron intensity as a function of *Q*

2 Q=4πsin(θ)/λ

where θ is the incident (and reflected) angle relative to the surface and λ is the neutron wavelength. Using the example of a single uniform film and a monochromatic source, as the incidence angle is increased, the path length between the top and bottom interface decreases, causing a pattern of constructive and destructive interference. or intensity oscillations with a period Δ*Q =* 2π/*T*, where *T* is the film thickness. For samples consisting of multiple layers, a more complex oscillation beating pattern is formed by interference from all of the interfaces.

Neutron reflectivity is a measure of the specularly reflected intensity minus the background intensity (at the same incidence angle) divided by the incident intensity, each at the same slit settings and normalized by the counting time per point. This data reduction was done using the Reflpak software.^40^ Data was collected using the Magik reflectometer at the NIST Center for Neutron Research.^41^ After precise instrument and sample alignment, specularly reflected intensity data were taken several times over the same series of *Q* ranges and compared to determine when the sample had equilibrated at each humidity. Data sets that agreed with each other within statistics were considered to correspond to an equilibrated sample, and any data sets prior to these (which differed statistically) were rejected. Further specular reflectivity was then taken at higher *Q* ranges, and the entire Q-range was typically repeated to ensure sample stability. Specular and background data were taken over a series of incident angle ranges to allow for data acquisition times of these ranges to be adjusted to ensure adequate counting statistics. Background data were taken by offsetting the detector to plus and minus 25% of the specular detector angle, except below an incident angle of 0.5°, where the detector offset was a constant plus or minus 0.25°. For specular and background measurements, incident slits were opened proportionally to the incident angle to maintain the same footprint of the beam on the sample. See the literature for further details.^12^

The reflected amplitude can be directly calculated using the Schrödinger equation for reflection from a barrier. Any arbitrary profile of SLD­(*z*) can be accurately modeled as a series of arbitrarily thin layers with uniform SLD across their thickness. Since the reflected intensity is the square of the reflected amplitude, the phase information is lost (unless reference layers are included to determine the phase^42^) and the NR data cannot be inverted to determine the SLD profile. However, because the NR can be exactly calculated for a given SLD profile, least-squares refinement can be used to determine the SLD depth profile from the NR data. In the slab model, each material layer is described by four fitting parameters: the complex SLD, thickness, and interface roughness, then the interface can either be broken down into arbitrarily thin uniform layers or approximated using the Nevot Croce approximation.^43^

Fitting was done with Refl1D^44,45^ and Bumps^46^ software using the differential evolution adaptive metropolis (DREAM) algorithm, which is a population-based Markov Chain Monte Carlo method.^47^ It determines the best fit within the designated set of parameter ranges, unlike gradient descent approaches that can be stuck in local minima of χ^2^ close to the initial fit parameters while missing a better fit further off. Because the number of layers in the sample was not known *a priori*, in order to determine the best fit to the data, numerous models with different number of material layers were attempted. In some models, the parameters of these layers were independent of each other, in others, they were related by a formula to allow for fewer fitting parameters, for example, multiple interface lamellae can be modeled as a damped oscillation.^13^ The Bayesian Information Criterion, BIC, was used to determine the statically best fit between models with different numbers of fitting parameters; however, nonphysical solutions or those inconsistent with parameters that are known to be similar in the other data sets were rejected. More details about the approach and specifics of fitting each data set can be found in the Supporting Information.

### Relative Humidity Control

Humidity was precisely controlled by using a system assembled from both commercial and custom-built parts. An argon carrier gas was passed at 0.003 L/s into a dewpoint generator (Li-Cor model LI-610) containing H_2_O, which was placed in a temperature-controlled enclosure. The humidified gas is then passed through a heated hose into a chamber with Al neutron windows. The chamber and an enclosure around the input flange were also temperature-controlled to prevent condensation of high-humidity vapor. The sample temperature inside the chamber was monitored by a Cernox sensor attached to the substrate and controlled by a Lakeshore 304 controller reading a Pt resistance temperature sensor using a resistive heater, both of which were attached to the substrate mount, which was also water cooled. Water cooling allowed the heater to operate at sufficient power to quickly compensate for small variations in heat load on the chamber and maintain a stable temperature that varies by less than 0.05 °C during the course of the roughly 24 h long neutron reflectivity measurement. The humidity was determined by converting the dew point to a vapor pressure, then to relative humidity at the sample temperature.^48^ It was also monitored by a sensor (Rotronic model HC2-S3H sensor and model HF53W XMTR controller), which agreed with the calculated RH to within uncertainty after correcting for the difference between the probe and sample temperatures, using the same vapor pressure formula.

For measurements under high humidity, the chamber and sample were equilibrated at the desired humidity (RH = 90%) for 1 day before the experiment, when possible. If not, the humidity in the chamber was monitored by the humidity sensor until it equilibrated. In all cases, the lack of changes in NR data for sequential runs was used to verify equilibration of the sample itself. For measurements at RH = 0%, the sample was dried in situ, in flowing Ar with a flow rate of typically (0.015–0.025 L/s) after bypassing the dew point generator. When the chamber RH reached 0.00% the sample temperature was raised to 60 °C or 120 °C at which it remained for 60 min or more at RH = 0.00% under flowing dry Ar. The sample was then cooled in flowing Ar to the desired temperature. The Ar flow rate was lowered but remained at levels that maintain RH = 0.00%

## Results

### Sulfonate and Fluorinated SAM Interface Structures

Neutron reflectometry data as a function of *Q* taken for the sulfonated SAM (S-SAM) and the fluorinated SAM (F-SAM) samples at both 90 and 0% RH are shown in Figure 2. While Monte Carlo-based least-squares fitting of the data to a variety of models provides definitive sample structures (i.e., the depth profiles of the SLD with uncertainty bands), it is useful to also demonstrate, when possible, the confidence in these fitted structures by pointing out what features of the data they are derived from. The highest frequency Kiessig oscillations, with a periodicity in *Q* that is inversely proportional to the film thickness, persist with a large amplitude out to high *Q*, indicating a film with uniform thickness and smooth interfaces. The broader peaks in the data (as opposed to the high-frequency oscillations) are associated with relatively periodic interface structures and have a peak width that is inversely proportional to the number of bilayer periods. The broad peak at *Q* ∼2 nm^–1^ has been identified with the presence of multiple lamellae at the interface with the substrate.^8^ The narrower peaks seen in Figure 2, for the humidified samples and the broader peak for the dried F-SAM (blue) are indicative of a multilamellar structure with more or fewer repeat units, respectively, while the much broader oscillation in the data for the dried S-SAM (brown) is indicative of a single layer at the interface.

**Figure 2:**
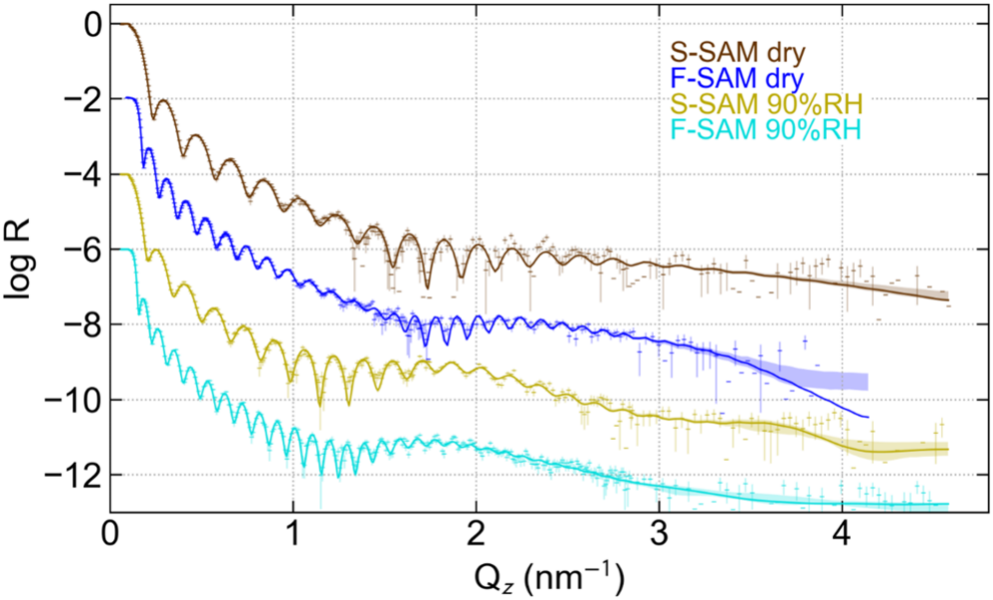
Neutron reflectivity data and fits for sulfonate and fluorinated SAM, both dry and humidified with H_2_O to RH = 90%.

Precise data fitting yields quantitative information in the form of the SLD depth profile through the thickness of the film, shown by the solid lines in Figure 3, along with an uncertainty band for each fit, corresponding to the 68% confidence interval, which is shown as shaded regions of the same color. Further details about the data acquisition and fitting procedures are found in the methods section, Supporting Information, and the literature.^12,13,16,17^ The roughly 1 nm thick structure that begins at *z* = 0, which is common to all samples, is the native oxide on Si. Above this layer at positive *z* values, the near-interface structure of the SAM and Nafion for each case differs significantly.

**Figure 3:**
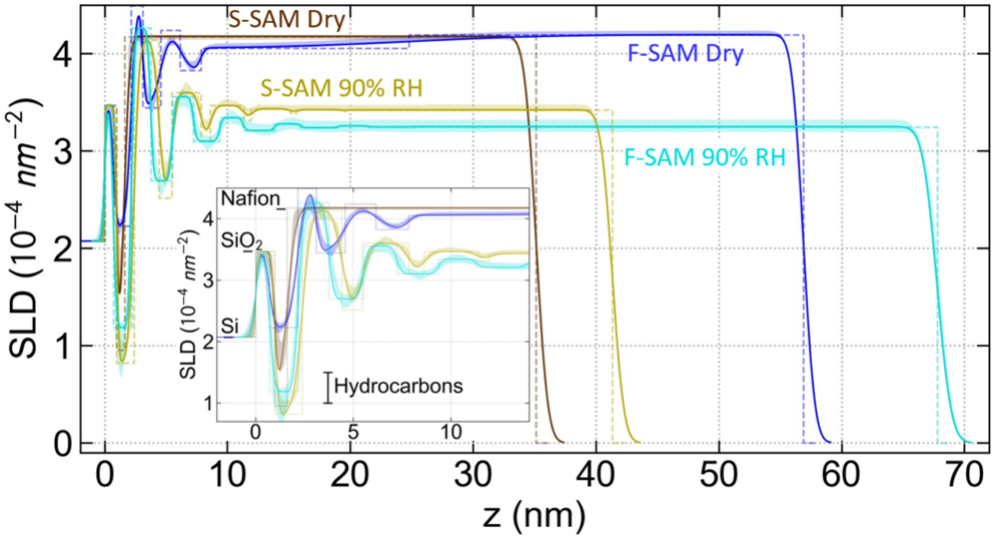
SLD depth profiles (lines) for the F-SAM and S-SAM samples dry and at 90% RH. The Si substrate is at *z* < 0 and the free surface is toward the right. The inset expands the interfacial region. Dashed lines represent the slab model without interface roughness.

Beginning with the S-SAM dried at 120 °C shown in brown, the layer immediately above the native silicon oxide has a thickness of 0.64 [0.53, 0.88] nm. (Throughout this manuscript, the numbers in square brackets refer to the lower and upper bounds to the 68% confidence interval for the parameter value obtained by the fit, in the NR and SLD depth profile figures the shaded bands represent 68% confidence intervals, reported χ^2^ values are the reduced χ^2^, and the error bars on data are one standard error.) The fitted SLD of this layer, 0.952 x10^–4^ nm^–2^ [0.64, 1.83] x10^–4^ nm^–2^, is consistent with a hydrocarbon, typically ∼1 x10^–4^ nm^–2^ to ∼ 1.5 x10^–4^ nm^–2^. Two versions of the SLD profile are shown in Figure 3. Dashed lines show the slab model, omitting the effect of the interfacial roughness between the layers. The best-fit model, including the interfacial roughness between the layers, is indicated by the solid lines. Note that large interface roughness, that is, intermixing between adjacent layers, causes the minimum of the profile, roughly 1.5 x10^–4^ nm^–2^, to be higher than the fit parameter indicated in the dashed curve. The thickness of this layer is nominally consistent with the ∼0.5 nm length of the tail group of the S-SAM (or ∼0.6 nm length of the S-SAM including the attaching Si). Given the agreement between both the thickness and the SLD, we attribute this low SLD layer seen to the right of the silicon oxide layer to the S-SAM. While the thickness of this layer is admittedly half of that needed to observe a full intensity oscillation in the observed Q-range of NR data (a rule of thumb for the minimum resolvable layer thickness is 2π/*Q*_max_ = 1.3 nm), we note that the presence of this interfacial S-SAM layer was required both to fit the shallow broad peak in intensity from *Q*∼1.5 nm^–1^ to ∼4.5 nm^–1^ and to provide needed contrast for the higher frequency oscillation. Each sample has a low SLD layer adjacent to the SiO_2_, which for simplicity will be referred to hereafter as the “SAM layer”, although it may also contain other materials, including water or Nafion moieties.

The best fit was found to have only this one SAM layer followed by the Nafion main layer with a thickness of 33.513 [33.306, 33.516] nm and the same SLD within uncertainty as dry Nafion (indicated in the inset of Figure 3). Here the term “main layer”, refers to the thicker uniform portion of the Nafion film that is not a thinner interfacial or surface layer or gradient at either the buried interface or the free surface. Attempts to include an additional layer to the model, which could become either an additional interfacial layer, a gradient in the noninterfacial Nafion layer, or a surface layer, resulted in a surface layer with slightly higher SLD than the main layer, but the SLD profile was not consistent with known physical constraints and was rejected. However, they did maintain a similar interface structure.

When the S-SAM was hydrated in RH = 90% H_2_O (yellow profile), the Nafion swells and the SLD of the main Nafion layer decreases to 3.423 × 10^–4^ nm^–2^ [3.407, 3.478] × 10^–4^ nm^–2^ due to absorption of water. This SLD corresponds to a water volume fraction of 0.1557 [0.144, 0.159] or λ = 5.69 [5.19, 5.83] (λ is the number of absorbed water molecules per sulfonic acid in the Nafion). The S-SAM layer also adsorbs water as indicated by an increase in thickness to 1.41 nm [1.38,1.49] nm and a decrease in SLD to 0.822 × 10^–4^ nm^–2^ [0.714, 0.997] × 10^–4^ nm^–2^. This layer thickness exceeds the length of the tail of the S-SAM (Si–C–C-C-SH) and therefore this layer must also include, to some extent, the sulfonic acid-terminated side chains of the Nafion. This first water-rich layer is also thicker than that observed for Nafion on SiO_2_ (on average 1 nm for the same total Nafion thickness and layer SLD)^9^ due to the presence of the S-SAM. In addition, multiple water-rich and water-poor lamellae form on top of the hydrated S-SAM layer with SLDs similar to those observed in Nafion adjacent to SiO_2_ surfaces for a sample of similar thickness.^9^ Presumably the mechanism is similar, with the sulfonic acid of the Nafion side chain attracted to the interface, this time to a similar compound, i.e., sulfonic acid in the S-SAM (rather than SiO_2_) and to the associated water during the initial spin coating process. As described previously, this segregation of the side chains to the planar interface serves as a template for a repeating pattern of water-rich and water-poor layers^8^, which involve the alternating enhancement of the two moieties of Nafion (fluorocarbon backbones and sulfonic acid-terminated side chains) with diminishing composition variation further from the interface.^13^ However, the water-rich layers on the S-SAM are much thinner than they are for Nafion on the other SAMs in this study and for Nafion on SiO_2_, possibly indicating less phase segregation of the side chains into those layers compared with the other cases.

Under the same 90% humidity, the F-SAM sample (Figure 3, Cyan) also has multiple lamellae at the interface. The F-SAM layer and the next 3 lamellae have SLD levels very similar to those found for the S-SAM, indicating similar water content in both the water-rich and water-poor lamellae. The main layer has a greater water uptake in the F-SAM sample (λ = 7.34 [6.67,7.71]) than in the S-SAM sample (λ = 5.69 [5.19, 5.83]), which is consistent with the effects of the greater sample thickness as previously reported;^9^ these films are still within the regime where Nafion thickness influences overall water uptake. Both the water-rich and water-poor lamellae approaching the main layer also have increased water content consistent with greater thickness, and the water-rich layers are also thicker, consistent with more water for similar amounts of Nafion side chain material.

It is notable that in the hydrated condition, the F-SAM layer has an SLD of 1.186 × 10^–4^ nm^–2^ [0.525, 1.233] × 10^–4^ nm^–2^ and thickness of 1.482 nm [1.177, 1.523] nm, similar within uncertainty to the S-SAM, and consistent with a high water content. This is counter to our initial hypothesis that the fluorocarbon tail of the F-SAM would both attract less water during spin coating since it is hydrophobic and would preferentially interact with the hydrophobic backbone of the Nafion, thereby inducing a fluorocarbon-rich (high SLD) layer at the interface. An attempt to fit the data with a fluorocarbon-rich (high SLD) layer at the interface resulted in much higher χ^2^ (1.360 vs 1.101 for the best fit) as described in the Supporting Information, indicating that the low SLD hydrated F-SAM layer has been firmly established. It appears, rather, that the interfacial layer contains a large portion of water in addition to the F-SAM. One explanation is that there is only a partial surface coverage by the F-SAM headgroups because the fluorocarbon tail, which is much longer than that of the S-SAM, may lay across the surface, blocking complete coverage by the headgroups. Then when Nafion dispersion is added during spin coating, the tails might lift from the surface, leaving some of the very hydrophilic SiO_2_ exposed and accessible to water and sulfonic acid side chain penetration before the postdeposition drying. Another explanation is that the presence of water in the Nafion dispersion cleaves some of the bonds between the F-SAM and SiO_2_ as seen for silanol;^49^ however, this is not likely since this effect does not take place for the neutral A-SAM sample. Instead, the decreased surface coverage of the F-SAM is supported by the fact that the water contact angle of the F-SAM is 60° whereas the advancing contact angle of highly fluorinated surface such as Teflon is 115.3°.^50^ The lower contact angle could have been caused by partial coverage. This exposed SiO_2_ would induce a multilamellar interface structure similar to that of Nafion on SiO_2_ alone. Note that the F-SAM tail, roughly 1.1 nm, or 1.5 nm including the headgroup, is not long enough to effectively span the 1.4815 nm [1.177, 1.523] nm F-SAM layer to interact significantly with the fluorocarbons in the Nafion backbone, unless the fluorocarbon backbones were also present in the F-SAM layer.

For the F-SAM sample dried at 60 °C, typical for studies of Nafion, and measured RH = 0% (blue curve Figure 3), the number of distinct lamellae is reduced from 6 (for the hydrated case) to 4. All lamellae decreased in thickness in the absence of water except the F-SAM layer. It has a thickness of 1.44 nm [1.32, 1.50] nm, similar to that when it is hydrated 1.48 nm [1.18, 1.52] nm, but has a much higher SLD. This indicates that water was removed, but the layer did not contract. These observations are similar to the effects observed for Nafion on SiO_2_ ^13^, which was attributed to insufficient mobility (at the 60 °C used for drying) to reorient the Nafion side chains spanning the first lamella and to fully remix Nafion’s sulfonate and fluorocarbon moieties of subsequent lamellae. Because the SLD of water and unfilled voids is similar, the significant increase in SLD indicates that other materials have moved into the layer. In addition, upon drying, the interfaces in general become broader, indicating some interdiffusion.

Unlike previous samples, a slight gradient in SLD is observed between these residual lamellae and the main layer (which, like the S-SAM, has an SLD equivalent to that of dry Nafion). The presence of this gradient is supported statistically. Furthermore, the gradient was selected in the best fit, even though models allowed it to be prevented if the SLD values or thickness would have fitted to values similar to the lamellae or the primary layer. Models that did not allow for the gradient layer had considerably worse χ^2^ and BIC values. The decrease in the SLD approaching the lamellae could be due to some residual water, a decrease in density, perhaps due to the presence of F-SAM molecules that were cleaved from the SiO_2_ surface during spin coating, or a systematic error in the data (for example, lateral inhomogeneities which are not included in reflectometry modeling theory). Further research will be required to resolve this issue.

### Amine SAM Interface Structures

Two amine-terminated SAM (A-SAM) samples, as described in Figure 1, were also studied. For the first of these samples, a Nafion film was spin-cast onto the neutral A-SAM-treated Si substrate, produced in a way similar to the S-SAM and F-SAM samples. This process preserves the –NH_2_ functionality of the terminus group. Neutron reflectivity data and best fits for this sample as prepared then hydrated with H_2_O at RH = 90% are shown in purple in Figure 4. Simple inspection of the data shows that unlike the F-SAM and S-SAM at high humidity, there is a complete *absence* of the high *Q* peak associated with multilamellar structures. The experimental reflectivity data are well fit to the model shown in the same color in Figure 5, in which, in addition to a surface layer, just two interfacial layers are required. The first, low SLD, A-SAM layer is similar to those seen on the F-SAM and S-SAM, although with a slightly lower SLD, 0.721 × 10^–4^ nm^–2^ [0.62, 0.79] × 10^–4^ nm^–2^ and thicker 2.133 nm [2.058, 2.178] nm, indicating the presence of more water. The second layer has an SLD very close to that of the hydrated main layer. While this layer improves the χ^2^ and BIC slightly (BIC is 8 lower), the improvements are within uncertainty and this second layer is only tentatively established. The fit in which this layer is not allowed is nearly identical otherwise (see the Supporting Information). A thin surface layer is seen in both of these fits with similar thickness (4.25 nm [4.00, 4.38] nm in the best fit) and SLD slightly above that of the main layer. This surface layer results in a BIC that is 33 lower than without it, strongly indicating its presence.

**Figure 4:**
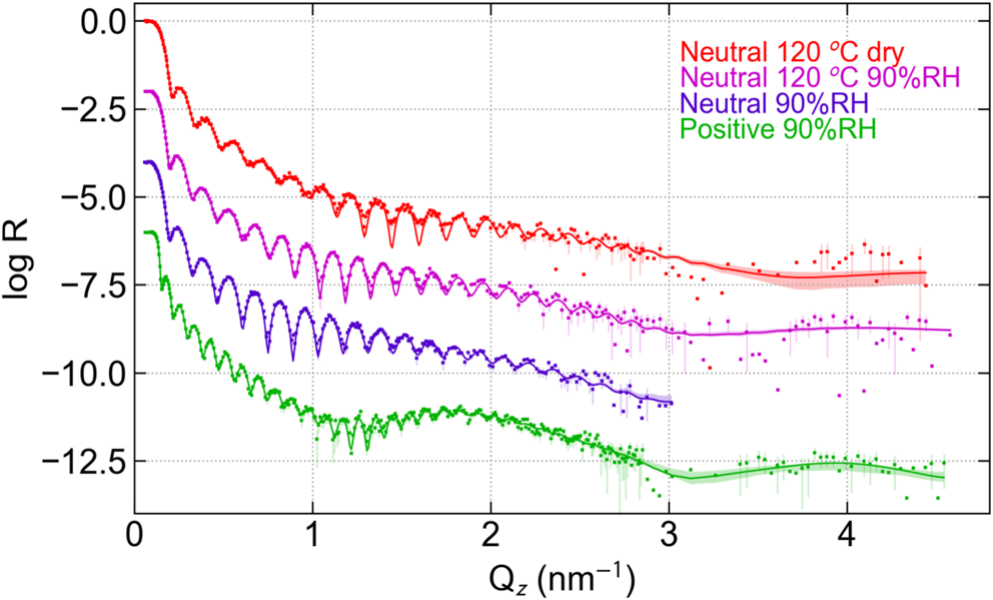
Neutron reflectivity data and best fits for the amine SAM, annealed (top 2 curves) and unannealed (bottom 2 curves). The top curve is taken at RH = 0% the other 3 were taken at RH = 90%. The top 3 curves are for a neutral A-SAM and the bottom curve is taken for a sample in which an A-SAM was created in the positive form by treatment in pH = 4. Only the positive A-SAM has a high Q peak indicative of a multilamellar interface structure.

**Figure 5:**
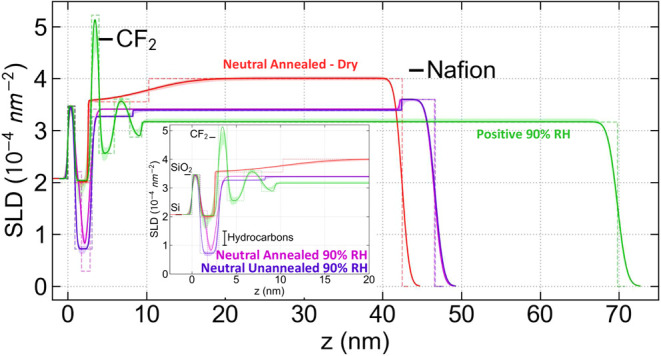
SLD depth profiles (lines) and 68% confidence intervals (shaded bands) for the neutral A-SAM and positive A-SAM samples, dry and at 90% RH. The Si substrate is at *z* < 0 and the free surface is toward the right. The inset expands the interfacial region. The SLD of various components of the sample is indicated by horizontal bars as labeled.

In order to investigate if there were possible interactions between the neutral A-SAM and Nafion that were either kinetically limited or thermodynamically hindered, the sample was annealed in situ at 120 °C for 60 min in Ar at RH = 0%. The annealed neutral A-SAM sample, when hydrated in H_2_O at RH = 90%, shown in magenta in Figure 4, also does not have the high *Q* peak associated with the multilamellar structure. Fits to the data (Figure 5) result in a model with a SAM layer with roughly the same thickness as before the annealing but with a slightly higher SLD, indicating less water than for the unannealed case. This A-SAM layer consisted of two separate layers that were required to fit the data well. The one adjacent to SiO_2_ was 0.838 nm [0.560, 0.925] nm thick with SLD = 2.21 × 10^–4^ nm^–2^ [1.97, 2.31] × 10^–4^ nm^–2^ followed by one 1.049 nm [0.988, 1.485] nm thick with SLD = 0.294 × 10^–4^ nm^–2^ [0.294, 0.959] × 10^–4^ nm^–2^. These two layers in effect modify the SLD profile of the SAM layer, indicating that it is less uniform than those for the other samples. In addition, the interfaces on each side of these layers are broadened, also seen to some extent for the S-SAM, indicating that a possible greater intermixing occurred due to the higher temperature annealing. The high SLD second interfacial layer tentatively seen in the unannealed case is no longer present. The main Nafion layer has the same SLD and thus water uptake as the unannealed sample. As in the pre annealed hydrated case, a 4.42 nm [4.30, 4.55] nm thick surface layer is present in the annealed sample with SLD slightly above that of the main Nafion layer, indicating a surface with less water uptake. This surface layer may be the fluorocarbon-rich, hydrophobic layer that has been reported in experimental^51,52^ and computational^53^ studies. While the presence of the surface layer in both cases provided a statistically significant improvement to the fits, it is also possibly an artifact of systematic error (see the Supporting Information) and has been seen in some but not all cases in other studies.^9^ It is of note that while the models did not require it, the total hydrated thickness of this sample was the same before and after annealing. This is also indicative that the water uptake was the same in both cases.

The annealed neutral A-SAM sample was also measured in the dried state before hydrating it. In this condition the SLD of the A-SAM layer increased considerably, indicating a loss of water, but with only marginal shrinking, similar to the interfacial water-rich layers of F-SAM sample and Nafion deposited directly onto SiO_2_.^13^ The neutral A-SAM layer had SLD of 1.995 × 10^–4^ nm^–2^ [1.88, 2.04] × 10^–4^ nm^–2^ similar within uncertainty to that of the F-SAM 2.228 × 10^–4^ nm^–2^ [2.07, 2.28] × 10^–4^ nm^–2^, but larger than that of the S-SAM 0.952 × 10^–4^ nm^–2^ [0.64, 1.83] × 10^–4^ nm^–2^.

In addition, there is a gradient in SLD between the dry annealed A-SAM layer and the main Nafion layer, with a larger SLD difference over a shorter distance than that observed for the dry F-SAM sample. This gradient was strongly required by the fitting in that it improved BIC by over 138 compared to models that did not have it. Models that allowed this gradient to be at the free surface and allowed a surface layer did not result in an improved fit for physically possible profiles. The SLD of the main Nafion layer increased, consistent with the removal of water, but (unlike the other unannealed samples, including the S-SAM also annealed at 120 °C) remained slightly less than that of dry Nafion. While annealing typically increases density by increasing crystallization, one possible explanation for the lower SLD is a lower polymer density after annealing at 120 °C, perhaps by aggregating ionic domains and locking in voids.^54^ The second possibility, retention of water, is not likely given the higher temperature used to dry the sample and the fact that it was maintained in a dry environment for the entire period during annealing through the end of the NR measurement. Both these explanations are not likely in that they are not seen in the case of the similarly annealed S-SAM. The large gradient is likely related to the lower SLD interface layer seen in the preannealed case.

A second sample (positive A-SAM) was prepared by soaking a separate A-SAM layer, prepared as before, in a buffer solution with pH = 4 for 30 min to protonate the terminal amine and impart a positive charge (−NH3^+^) prior to spin-casting the Nafion film onto the surface. This positively charged surface clearly resulted in a large, high Q peak in the NR data (Figure 4 green curve), indicative of a strong multilamellar structure near the interface. This was verified by the best fit, in which there are 5 alternating high and low SLD layers indicating water-poor and water-rich lamellae, respectively, similar to the number of lamellae found in Nafion on SiO_2_ of similar thickness and RH.^9^ This structure is very different from that seen for the neutral amine SAM in which only one water-rich layer occurs at the interface with SiO_2_, both as-prepared and after annealing. Therefore, treating the amine SAM to have a positive charge changes the hydrated interface structure from a single water-rich interfacial lamella (the SAM layer) to multiple lamellae at the interface.

The multilamellar structure is different on the positive amine sample than on SiO_2_ and F-SAM, suggesting a different interaction between the substrate and Nafion. The bilayer period of the multilayer structure in the positive A-SAM is slightly larger than that found for the hydrated F-SAM and S-SAM, as well as SiO_2_. The first water-poor layer has an SLD greater than the SLD of dry Nafion but is equal to the SLD of the fluorocarbon backbones of Nafion within its relatively large uncertainty. This implies that not only is most of the water removed from this layer, but the remaining material also excludes the sulfonic acid terminating the side chains. This phase separation of the Nafion moieties is similar to what was observed for Nafion on SiO_2_, however, in that system some water (∼3% by volume) also resided in this layer.^13^ The best fit to the data also indicated that more water is in the main Nafion layer than for the neutral amine, consistent with more water in the lamellae as observed previously.^9^

## Discussion

While some features, such as the surface layers in the hydrated neutral amine SAM, and the second interfacial layer in the unannealed one, are not definitively established, other structures are strongly supported by fitting and verified by significant improvements over fits to models that exclude these features. From these well-established structural features, observations about the ordering of Nafion deposited on self-assembled monolayers can be made.

While it would be expected that the F-SAM tail would interact preferably with the fluorocarbon chain in the Nafion backbone, resulting in a high SLD layer adjacent to the SiO_2_, instead, the results show a low SLD layer (followed by a high SLD layer), indicating that the sulfonic acid side chain interacts with the substrate. It is well established here that the SLD of this layer, when dried, is well below that of Nafion and fluorocarbons. When hydrated, the SLD of this first lamella decreases to a low value relative to the dry state, indicating the presence of a considerable amount of water in that layer. This behavior is consistent with the sulfonic acid-terminated side chains of Nafion being exposed to some of the underlying SiO_2_, implying partial coverage by the F-SAM head groups. This could occur if during deposition of the SAM some of the F-SAM tail, which is longer than that of the S-SAM, is oriented parallel to the SiO_2_ surface, blocking access of additional headgroups to it. When the Nafion is added, these tails could then lift from the surface into the Nafion allowing access of Nafion’s sulfonic acid-terminated side chains to bind to the SiO_2_, which induces the multiple lamellae, starting with water-rich layers, as for Nafion on SiO_2_.^8^ Upon drying the F-SAM sample, like Nafion on SiO_2_ the first lamella retains its hydrated thickness within uncertainty and multiple residual lamellae remain, which indicates that there is insufficient mobility to overcome the stronger bonding of the sulfonic acid to SiO_2_, which is responsible for these structures. This may be because the temperature used to dry the samples is well below the α transition temperature.^55^

The water-rich layers of the multiple lamellae of the hydrated S-SAM sample are thinner than for hydrated Nafion on the F-SAM, the positive A-SAM, and on SiO_2_. This could be explained by a lower concentration of the sulfonic acid and consequently water in those layers, with more of the sulfonic acid remaining mixed with the fluorocarbon backbones in the higher SLD lamellae than in the other cases. Upon drying the S-SAM sample, the multilamellar interfacial structure is no longer observed. With fewer sulfonic acid-terminated side chains associated with the lower SLD lamellae, remixing would be easier. The lack of residual lamellae was also observed for the Nafion on hydrophilic organosilicate glass, which had a contact angle similar to that of the Si native oxide.^23^ Film thickness did not play a role in this comparison since the studies on the native oxide covered the thickness range in this report and the OSG sample has a thickness (∼55 nm when dry) similar to the F-SAM, which showed a different structure.

Two factors explain the loss of multiple lamellae upon drying the S-SAM sample. The first is the weaker interaction of Nafion with the substrate, i.e., interactions between sulfonic acid groups of Nafion with the SAM rather than bonding to the SiO_2_.^28^ The hydrated S-SAM layer and F-SAM layer are similar in thickness, 1.4050 nm [1.379, 1.489] nm and 1.4815 nm [1.177, 1.523] nm, respectively, slightly thicker than the first water-rich layer (0.88 nm to 1.23 nm) for Nafion films of similar dry thickness (<60 nm) on SiO_2_. However, the S-SAM molecule length is shorter than this thickness, indicating that this layer likely also contains some of the sulfonic acid-terminated side chain of the adjacent Nafion. Upon drying, the S-SAM layer decreases in thickness, unlike the F-SAM layer and the first lamella of Nafion on SiO_2_. In the latter two cases, the retained thickness is likely due to the inability of the Nafion side chains (which bridge the layer and bond to the SiO_2_) to collapse. Therefore, the sulfonic acid of the S-SAM and Nafion interacts with each other within the SAM layer rather than the Nafion sulfonic acid side chains bonding to the substrate SiO_2_. This gives it the mobility to reorient, allowing the S-SAM layer to collapse and decreasing the phase segregation of Nafion at the substrate (which may then propagate outward). The OSG surface may also result in weaker bonding with sulfonic acid on Nafion’s side chains, compared to SiO_2_, which may also be the reason for a similar effect in that system.^23^ This weaker interaction likely also resulted in a smaller degree of phase segregation at the interface, which would also decrease the amount of phase segregation prorogating away from the interface.

The second factor that likely influences the elimination of the multiple lamellae upon drying is the thinner water-rich layers in the multiple lamellae for the S-SAM sample compared to those of F-SAM and Nafion in SiO_2_. This would require shorter distances for material to move, allowing greater intermixing upon drying. Also, these thinner sulfonic acid and water-rich layers in the hydrated state indicate that there is less phase segregation of the sulfonic acid from the fluorocarbons in the multiple lamellae. This broader distribution of the sulfonic acid may involve structures that better bridge the two regions and provide greater mobility of the moieties of Nafion upon drying, resulting in more complete intermixing and the elimination of the multiple lamellae.

Most importantly, the amine SAM can be modified to produce either of two very distinct interfacial structures under hydration, even though the hydrophobicity is the same for both A-SAM layers. For the untreated neutral A-SAM at RH = 90%, a single water-rich A-SAM layer is formed at the interface. Above this, there may be an additional layer that contains slightly more water than the remaining main Nafion layer. Annealing this sample at 120 °C did not result in a substantially altered hydrated structure. However, when the amine SAM is treated to create a positively charged terminal amine, by soaking the A-SAM layer in a pH = 4 solution before the deposition of the Nafion, the resultant positive A-SAM is observed to have a very distinct multilamellar structure, associated with four water-rich and water-poor layers above the positive A-SAM layer. Thus, the hydrated Nafion interface structure can be switched from having a single water-rich layer to having a multilamellar structure by the application of a pH = 4 environment to produce a positive charge in the amine SAM layer.

## Conclusions

This work confirms the atomistic nature of Nafion bonding via the interactions of Nafion’s sulfonic acid groups with the terminal amine of a 3-Aminopropyltrimethoxysilane (APTMS) molecule, demonstrating that interactions other than hydrophilicity can induce multilamellar interface structures in Nafion and may lead to numerous practical applications. The use of SAM layers can provide tunable control over the interfacial structure of Nafion as a function of RH, which may be useful in controlling the ionic conductivity both parallel and perpendicular to the interface, which could enable the design of new devices and improve the efficiency of the catalyst layers. For example, coating the carbon black particles with positive amine SAM (which induces multiple lamellae in Nafion at high RH) could induce transport parallel to the surface, thus increasing fuel cell efficiency or lowering the amount of Pt loading that is required.

By utilizing this ability to control the interaction of Nafion with the A-SAM by controlling its charge, it may also be possible by writing patterns of amine charge laterally across a surface to similarly write lateral patterns of multiple lamellar structures (which lie parallel to the surface) and thus write regions of larger in-plane and smaller through-plane conductivity.

The F-SAM (and perhaps other long-chain SAMs) produces a structure similar to those observed on the underlying substrate, in this case SiO_2_, creating multiple lamellae at the interface at both RH values studied, 0 and 90% (and likely greater), presumably due to partial coverage of the F-SAM layer. The S-SAM, a short-chain SAM, exhibits multiple lamellae only at elevated humidity, converting at 0% RH to a single low SLD layer, which contains the SAM itself. It can be a powerful tool to induce the multilamellar structure only at high RH onto any surface, independent of its hydrophilicity, i.e., to allow switching of in-plane transport with humidity. The neutral A-SAM, also a short-chain SAM, does not induce multiple lamellae at any RH. By controlling the ratio of a mixture of S-SAM and neutral A-SAM, the surface interaction strength for inducing lamellae can be continuously controlled.

SAMs may also be used to tune how additive nanoparticles interact with the separate moieties of Nafion, resulting in the possibility of placing them in, near, or away from the ionic domains. Bulk properties of Nafion may be modified by placing the desired functionalized macromolecules into the ionic domains in bulk Nafion by attaching them to positively charged amine groups. (Or for a lower interaction strength, sulfonate groups can be used.) This approach may also be used to tag the ionic domains with active molecules, such as dyes or NMR active material, to enhance signals of various probes, enabling better structural characterization. Polymer chains or stars with positive amine groups at all end points may be used to link ionic domains in Nafion.

## Supplementary Material



## Data Availability

The data that support the findings of this study are openly available in the NIST Center for Neutron Research public data repository at the following sites. A key to date set names and dates is given below: 10.18434/T4201B?urlappend=cg1/201101/Dura/HPS/ S-SAM, Humidified sfvh1 2011–01–18 S-SAM, Dry sfvh2 2011–01–20 F-SAM, Humidified flvh1 2011–02–14 Neutral A-SAM, Humidified am1vh1 2011–01–18 Neutral Annealed A-SAM, Dry am1vh2 2011–01–19 Neutral Annealed A-SAM, Humidified am1vh3 2011–01–20 Positive A-SAM, Humidified am2h1 2011–02–14 10.18434/T4201B?urlappend=magik/201503/16349/data/ F-SAM, Dry flvh2 2015–04–28

## Notes

Additional details of the approach to data fitting, and discussion of relative uncertainties (PDF)
