# Supplementary material for: Controlling Polymer Electrolyte Interfacial Morphology through Chemical Interactions
Source: Chem Mater. 2025 Aug 18;37(17):6921–31. doi: 10.1021/acs.chemmater.5c01738 (PMC12424123; doi:10.1021/acs.chemmater.5c01738)
Supplement: Supplementary file 1 [file cm5c01738_si_001.pdf]

Supporting Information for:

Controlling Polymer Electrolyte Interfacial Morphology through Chemical Interactions

Joseph A. Dura*[a], Sangcheol Kim[b], Kirt A. Page[c], and Christopher L. Soles[d]

[a]NIST Center for Neutron Research, Gaithersburg, MD 20899, United States

[b]Wiss, Janney, Elstner Associates, Inc, Northbrook, Illinois 60062, United States

[c] Av Inc., 4401 Dayton-Xenia Rd., Dayton, OH 45432, United States;

Cornell High Energy Synchrotron Source, Cornell University, Ithaca, NY 14850, United States;

Materials and Manufacturing Directorate, Air Force Research Laboratory, WPAFB, Dayton, OH 45433, United States

[d]NIST Materials Measurement Laboratory, Gaithersburg, MD 20899, United States

* Email: Joseph.Dura@nist.gov, JoeDura0@gmail.com

S1 Neutron Reflectometry Data Fitting

S1.0 General Description

Nafion thin films can have a complicated depth profiles including multiple interfacial lamellae, followed by a non-interfacial layer (typically a thicker uniform "main" portion of the film[1]), potentially followed by surface layers[2]. The structure may or may not also have gradients of water content or composition. The number of layers in the sample is not known ahead of time, nor can it be determined by simple examination of the data, it must be determined by trial and error, through fitting the NR data to different models and comparing the quality of fit between models using the BIC parameter[3] and by validating the structures and parameters to be consistent with known material properties. Therefore, a careful, thorough, and systematic approach to fitting the reflectometry data is needed to determine the profile that best represents the sample - without asserting spurious features. The approach used for this paper is described and explained below.

Refl1d[4,5] using Bumps[6] calculates NR[7] from a SLD profile determined from model parameters (typically a slab model although other SLD profiles such as freeform curves are also available). A slab model is one that fits each model layer to a uniform complex SLD, and thickness with interface profiles representing roughness and interdiffusion. Interface profiles that are error function shaped (i.e. from interdiffusion) are very well represented by the Nevot-Croce approximation.[8] Other functions can be modelled by dividing the non-uniform region near the interface into a series of very thin uniform layers whose SLD follows the desired interface shape. Ref1d using the Differential Evolution Adaptive Metropolis (DREAM)

algorithm applies a Markov Chain Monti Carlo approach to generate the probability distribution in parameter space to determine the global best fit of the parameters of that model to the data.

In complicated models with k fitting parameters the probability "landscape" or χ^2_{norm} (or inverse of likelihood) as a function of k-dimensions has many local minima, some of which can have similar likelihood. (Here χ^2_{norm} is the χ^2 value of the fit, normalized by dividing by (n-k) where n= the number of data points such that for an ideal fit $\chi^2_{norm} = 1$. In the main paper χ^2_{norm} values are reported but for simplicity simply referred to as χ^2.) In fact, there can be two or more symmetry related fits that both produce exactly the same reflectivity profile. Therefore, it can be difficult to find a fit to the data that accurately represents the actual SLD depth profile of the sample. Typically, this is the globally lowest minimum of χ^2_{norm}, but for two solutions that are statistically similar, this requires determining the better solution, often the one that is either more physically meaningful or consistent with the other data sets or known properties. Refl1d also determines uncertainties of not only the parameters but also SLD profiles and the theoretical best fit NR curve. Further advantages are that Refl1d can show correlations between parameters. More info on Refl1D can be found in the references[4,5,7]. However, for the purpose of this description the important points are that, unlike gradient descent approaches, the global best fit to a given model can be found, or if two fits are of similar likelihood, that can also be indicated.

Several modeling approaches can be used to determine the SLD profile. The Independent Layer, IL, model uses the slab approach with each layer having fitting parameters (complex SLD, thickness and interface roughness) that are not related to the other layers. The Damped Oscillator, DO, model determines the SLDs of the lamellae from the Nafion volume fraction, which is determined separately for the water rich and water poor lamellae as an exponential decay with two fitting parameters for each set, i.e. the volume fraction of the first water rich or poor layer and a separate decay rate for water rich and water poor lamellae. Similarly, the thicknesses of the alternating water rich and water poor layers, indexed by m, are fit to separate functions with a geometric rate of increase, $T_m = T_0 \gamma^m$. Thereby an arbitrary number of lamellae can be described with 8 fitting parameters. These two models were found to effectively describe the data [3]. Additional models can be used, for example those where the SLD profile is determined by a spline function. Or hybrid models can combine several approaches for different regions of the sample. A given model is defined by both the approaches that are used to determine the SLD profile, but also by the range of parameters investigated. For example, two IL models with different numbers of layers are separate models, but also two IL models with the same number of layers, but different ranges for fitting parameter(s) can also be considered separate models.

Within a model, the best fit is the set of parameters with the lowest χ^2_{norm}. However, a better χ^2_{norm} might be obtained by adding more fitting parameters, for example adding another layer in the slab model approach. If the more complex model can reproduce the SLD profile of the less complex model, then the χ^2_{norm} should be at least as low as for the less complex model. So as parameters are added, χ^2_{norm} should monotonically decrease or level off. These improvements however could just be that the random variations of the data are fit better, resulting in meaningless features in the SLD profile. The Bayesian Information Criteria, BIC, is used to determine if the improvements are statistically significant, or if they just happen to fit the statistical noise better. In the BIC there is a penalty for more parameters.

$$\mathrm{BIC} = (n-k)\chi^2_{norm} + k\ln(n) \qquad \text{Eq S1}$$

Where n is the number of data points and k is the number of fitting parameters. For typical systems where statistical error dominates, a BIC difference of 6 is considered significant[9] however this cutoff was chosen somewhat arbitrarily, and there are several systematic errors not included in the reflectometry models, so a somewhat larger value may be used. The BIC is useful for determining which of similar models is better, for example those where adding parameters can reproduce the same SLD profile as without them, but should be used with caution when comparing less related models. Models should also be rejected if they produce physically impossible SLD profiles, such as those with discontinuities in SLD profile, or contain SLD values that are not attainable by the materials in the sample or have other parameter values that are not consistent with known parameter values, or values were determined by other fits.

While these statistical approaches are useful there might also be systematic errors in the experiment (i.e. aspects of the reduced reflectivity not described by the model), for example in-plane non-uniformities of the lamellae or other layers, but also including less likely causes such as slight changes to alignment, sample changes during measurement, or things that are not included in the model for example roughness profiles that are not described by the error function, or in the scattering theory such as off-specular scattering or sample warp. Fitting data with systematic error can at times lead to SLD profiles with features that are not present in the sample. So, when unexpected features are present it is best to systematically confirm them for example on different samples, or with complementary measurements.

Furthermore, in demonstrating that the best fit to the data is also an accurate fit, it must be shown that the sets of models that were used have the flexibility or breadth of parameter ranges to completely allow for all possible solutions. That is, in a slab model do all the layers have sufficiently wide SLD range to capture any possible variations in density, porosity, composition changes due to intermixing with adjacent materials or solvents? Do the thickness ranges similarly consider all possibilities? Occasionally a model with one layer corresponding to each of the expected layers is found to provide a poor fit. When adding additional layers to account for interfacial reactions or gradients, are the SLD and thickness ranges sufficiently large to allow the new interfacial layer to reside between any two original layers, or are each possible location separately tried? Are the thickness ranges sufficient to allow for any ratio of thicknesses for a given material layer to be modelled as either two layers or a gradient? An unrealistic general approach would be to let the thickness of every layer to range from ~0 to a value significantly more than that expected for the entire sample. And similarly, to allow the SLD range for all layers to include all possible SLDs for any of the material present including considerations for a wide range of density and porosity variations. The problem with that approach is that an extremely large parameter space must be explored, resulting in many unphysical symmetry related solutions, or fits that never converge to a single solution.

S1.1 General approach to fitting.

Each data set was initially fit independently to numerous models to determine the best fit. However, in some cases this was made difficult by the large number of parameters that were required. Therefore, because the SiO_2 layer will not change with humidity or the modest temperatures used to dry the samples, a strategy was chosen in which the SiO_2 layer's fitting parameters for each sample were determined from one humidity, then fixed at those values for the other data set. In most cases the

dried condition was simpler to fit and was therefore used to determine the SiO_2 parameters which were then held fixed for the humidified condition (which, with more layers, had in general more fitting parameters). However, for the FSAM, the humidified data set was easier to fit and was used to determine the SiO_2 parameters.

Three fitting approaches were used. The IL and DO slab models were mainly used, although for the dry F-SAM data some models included spline fits to certain regions to explore if more complicated structures could better fit the data. Some variations to this approach to achieve a consistent model to the data were pursued as described below.

S1.2 Variations in SiO_2 top interface width

It was found that the interface above the SiO_2 was wider than typically observed for native oxides in the best fits of two of the samples that were used to determine the substrate parameters. These were the neutral amine SAM (8.9Å), and to a lesser extent the FSAM (3.5Å). To check to see if interface widths that are similar to the other samples were statistically equivalent, re-fitting was done. For the neutral amine sample two solutions were originally found, the better one had a thicker (15 Å) and rougher native oxide. To examine the other solution, the thickness was limited to a range of 5 Å to 13 Å. The new fit had a native oxide thickness and interface width consistent with the other samples while raising the BIC by only 1.73. Therefore the new fit was preferred for consistency with the known native oxide structure and the other samples. Similarly, for the FSAM, a fit similar to the best fit was run but with the fitting range for the native oxide relative interface width set to an upper limit of 0.3x it's thickness. This produced a similar fit, with an increase in BIC of only 1.48, again favoring the consistent SiO_2 thickness and interface width across all samples. For the final fits the SiO_2 interface widths were as follows S-SAM, dryfit3(1.50Å), F-SAM (2.05Å), neutral A-SAM (2.18Å), positive A-SAM(1.50Å).

S1.3 Other structures specifically tested

Some literature[10,11] has suggested that there might be a thin fluorocarbon rich layer at the surface of Nafion in some circumstances. This was specifically tested for all of the data sets, by fitting to models that included a surface layer. In some cases the fit with a surface layer had a better BIC and in other cases a worse BIC. However there was not a consensus that the surface layer exists on all samples. So at this point is it considered possibly a feature or possibly a result of systematic error. Other structures that were specifically tested were unusual or unexpected features like gradients, a high SLD in the first water poor lamella in the positive amine fit, and a high SLD layer adjacent to the SiO_2 for the Fluorinated SAM where one might expect a high concentrations of fluorocarbons if the SAM was concentrated at the interface.

S1.4 Details of Fitting the S-SAM data sets

Due to the relatively simple structure, consisting of only two layers above the native oxide, only three independent layer model fits were required to find the best overall fit the dry S-SAM data with a considerably low χ^2_{norm} = 1.279. The fitting ranges of the SAM layer thickness (4 to 30Å) and SLD (0 to $6x10^{-4}nm^{-2}$) were large enough to allow any physically allowable solution for this layer. Adding another layer and setting the ranges of the upper 2 such that they could represent either an additional interfacial layer, a gradient in the non-interfacial Nafion layer, or a surface layer, resulted in a fit that included two

similar local minima, shown in Fig S1 (top). Both solutions (with either high or low SLD surface layers) are seen in the uncertainty bands of this fit's SLD profile.

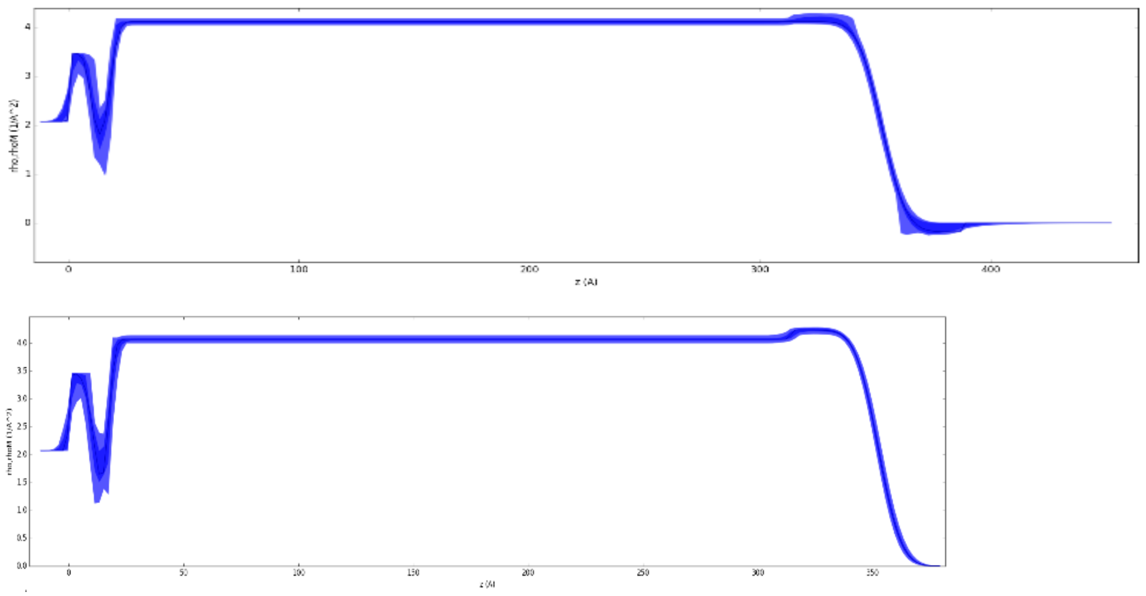

Figure S1 Fits to the S-SAM in zero humidity. (top) SLD profile of the fit with both solution in the uncertainty bands (bottom) fit that limited the surface layer SLD range to 0.05 to 7 and further refined with epsilon initialization.

The best fit of one of these had a 3.5nm thick surface layer with an SLD slightly below 0, which is not physically possible. To isolate the other solution, the fitting range of the SLD of the surface layer had a lower limit of 0.05 and was further refined. The result was a fit with an SLD profile (Fig S1 *bottom*) that included a 3.8nm thick surface layer with an SLD which is roughly equal to the theoretical value for dry Nafion SLD. In this fit the SLD of the non-interfacial Nafion was less than the bulk value corresponding to a Nafion Volume fraction of 0.9915, vs 1.0045 [0.997, 1.007] for the model with no surface layer. While the BIC of both these surface layer fits (431.4 and 431.7) are much less than (471.0) for the best fit, Fit #3, without a surface layer (which was used in the main text) they also have an SiO_2 roughness of 3.28 Å which was inconsistent with the rest of the samples and was rejected. Because the two surface layer fits have similar BIC and thickness and deviation in SLD from the surround, it may be possible that the surface layers may be present to compensate for a systematic error. With the current data we cannot definitively say whether or not a surface layer exists, and the fit without it was selected as the best fit due to compatibility of the SiO_2 layer. Regardless, both models with and without surface layers have very similar SAM structure and only one water rich layer - which is the point of this article.

For the Hydrated S-SAM, initially the DO model with an additional layer for the SAM provided an excellent fit with χ^2_{norm} =1.155. The SLD and thickness for the additional layer for the SAM had the same large range as for the dry sample. By allowing such a large range of SAM layer thickness, any profile, with oscillations starting at high SLD or starting at low SLD could be achieved. It fit to a thickness of 2.08A, near the lower limit of the fitting range, and an SLD similar to that of the first water rich layer,

and thus demonstrated that the first lamella had a low SLD and could be described as a single layer which included the SAM headgroup. Adding another Nafion layer near the surface and setting the ranges of the upper 2 layers such that they could represent either an additional interfacial layer, a gradient or a surface layer, resulted in a similar profile with a thin low SLD, high roughness, surface layer, that essentially modified the surface roughness profile, but was rejected due to an increase in BIC. Due to the large number, 5, of distinct interfacial lamella in addition to the non-interfacial layer, it was likely that an IL model would not achieve better BIC since it would require more fitting parameters, and a χ^2_{norm} better than the already low best fit of 1.155. However, to verify this, a 7 Independent layer model was also fit to the data. χ^2_{norm} was slightly lower and the interfacial lamellae profile was similar to the original best fit, indicating that it found the same solution. However, because of the additional fitting parameters the BIC was higher, so this fit was rejected.

The extra layer for the SAM had been included to allow flexibility of letting the initial lamella which contained the headgroup to differ from the Damped Oscillation profile. To test if this was needed the extra HG layer was removed. In this fit, the χ^2_{norm} decreased slightly, to 1.132, and with 3 fewer parameters the BIC decreased substantially to 438.86 providing the overall best fit to the data set.

S1.4 Fitting the F-SAM data

The F-SAM was the one exception where the SiO_2 parameters for this sample were determined from fits to the hydrated case instead of the dry data. Various fits to the hydrated case produced a consistent profile through the SiO_2 layer, whereas the dry case produced a variety of SiO_2 thicknesses which depend on the model used. The hydrated data was fit to DO models both with and without (fits 1 and 2) a separate layer for the SAM. When the SAM layer was included, models were tried with the series of DO layers beginning with either with a water rich layer (fits 3,4,5,6) or starting with a Nafion rich layer (fit 7). However, the profile was similar in all cases, with a low SLD layer adjacent to the SiO_2 followed by roughly 3 more oscillations.

In the hydrated state, the Fluorine SAM data consistently fits to a structure with an 8Å SiO_2 layer followed by a ~15Å water rich SAM layer, a ~13Å Dry Nafion layer followed by 3 more layers with damped composition oscillation. No models indicate a general gradient in the remainder of the film. The same structure is reproduced in various models, the simplest of which (fit 1) includes a Damped Oscillator on a fixed SiO_2 Layer (15 parameters). Two variations support the 8Å SiO_2 thickness. When 3 parameters were added to fit the SiO_2 thickness and surrounding interfaces, the SiO_2 layer is fit to 7.1A thickness (Fit2). When a separate layer is added to a 4.44A fixed SiO2 layer (again 18 parameters) the separate layer fit to an SLD similar to the SiO_2 to for a total thickness of 8.01A (Fit3,4). This fit also indicates that the thickness evolution of the water rich layers is less important than the SiO_2 thickness in determining χ^2_{norm}. Repeating this (Fit6) with a 14.8 Å fixed SiO_2 thickness provides a significantly worse fit.

The best general fit (fit 2) resulted in a SiO_2 interface width that was larger than for the other samples with a broad distribution of values. Refitting (Fits 8 and 8b) with this parameter limited to below 0.3 times the layer thickness (to make it consistent with the other samples) resulted in an almost identical profile to within uncertainty and negligible increase in BIC, (by 1.48). This was ultimately the best fit.

Adding a surface layer to the DO model resulted in a 67Å thick surface layer with SLD almost identical to the non-interfacial Nafion layer and an increase in BIC of 11.3. Also trying an IL model with SAM SLD range {0,6} to allow either a high or low SLD adjacent to the SiO_2 resulted in a profile with low SLD adjacent to the SiO_2, a χ^2_{norm} equal within uncertainty to the best fit and an increase of BIC of 34.2 Neither can be considered the best fit.

Because one would expect that the FSAM would induce a fluorinated and thus high SLD layer adjacent to the substrate, another model (Fit 13) was tried that forced the SLD of the SAM next to the SiO_2 to be high by limiting its SLD to the range {3.5,7}, in this case χ^2_{norm} increased considerably to 1.360 increasing BIC by 123.5 relative to the best fit This strongly supports the counter-intuitive idea that the Fluorocarbons of the SAM do not produce a high SLD layer near the surface to which they were applied.

The data for the dry Fluorinated SAM was the most difficult to fit well. Initially 47 fits which allowed for the SiO_2 layer parameters to vary were run, before fixing these parameters at the best fit for the humidified data set on the same sample. Numerous approaches were used, including IL models with 1-4 interfacial layers between the SiO_2 and the thick non-interfacial Nafion layer. Some of these models were simplified by subdividing one of the parameter ranges into to subsets and testing each separately (to help the model to converge when there were many fitting parameters). Some models allowed the outermost layer to be thick enough to represent a gradient in the non-interfacial layer or a surface layer (fit 17). Fit 36 found a 39Å thick surface layer with very slightly higher SLD than the bulk, indistinguishable from the main layer based on its 95% confidence interval, and χ^2_{norm}=2.055. Additional approaches included: one slab plus 1 or 2, 4 node freeform regions; 2 slabs plus a 2 node freeform; 3 slabs plus a constrained 3 node or 4 node freeforms; 3 or 4 slabs plus 0 node (i.e. a linear gradient of variable thickness); 4 slabs with 3 or 4 linear interfaces; DO with and without a separate independent SAM layer. The best fit (Fit 47) at that point had χ^2_{norm} = 1.226 and BIC=540.5 for a DO model with a separate layer for the SAM layer in which the Lamellae beyond the 5th from the SAM merged to form a gradient in SLD roughly equal in thickness to the uniform layer above it that had slightly higher SLD. However it had a structure that is likely unphysical with a 10.8A SAM layer followed by an extremely thin, 4A water rich layer with sharp interfaces, and an inconsistently very rough SiO_2 layer, so it is rejected in favor of a later model (fit #64) with similar χ^2_{norm} and BIC=556.5

After fixing the SiO_2 parameters to the best fit of the hydrated FSAM (starting with fit #48), both IL models (including those that allowed a Surface layer) and DO models were used. The best fit (#64) had 7 independent layers on top of the Fixed SiO_2, the last two of which formed a gradient with SLD very slightly increasing away from the substrate. It had no structures that were non-physical and χ^2_{norm} = 1.232(79) BIC=556.5, considerably better than others with the fixed SiO_2 values and a χ^2_{norm} equal within uncertainty to the best fit which fitted the SiO_2 parameters. Subsequently, models were run to specifically test for a high SLD fluorocarbon layer adjacent to the SiO_2, by restricting the SLD of that layer to a range {4,8}, resulting in a χ^2_{norm} =1.745 and BIC=729, clearly showing that there is not a fluorocarbon rich layer adjacent to the SiO_2 contrary to what would be expected if the FSAM were to remain localized where it was initially deposited before the Nafion was added.

S1.5 Fitting the three neutral A-SAM data sets

The dry (annealed) neutral amine data was used to determine the SiO_2 parameters for the other two data sets on this sample. Independent layer models were used in all cases. Originally the best fit included a low SLD SAM layer on top of the SiO_2 then a main Nafion layer. Next another layer was added that fit to an 8nm interfacial layer with slightly lower SLD than the main layer which together formed a gradient with lower SLD toward the interface. Because BIC=525.06 for this model was a considerable improvement over the model without the gradient layer (BIC=663.34) the gradient layer is considered a significant feature of the sample.

However, this fit has a SiO_2 layer that was thicker and rougher than the other samples. In order to test if those SiO_2 parameters were strongly required, it was refit with a model that restricted the SiO_2 thickness to a range that is compatible with the other samples. Since the roughness and thickness were correlated in the original fit, with two local minima of χ^2_{norm}, a thickness range limit was sufficient to select the local minima with both thinner and smoother SiO_2. This fit (#6) has a χ^2_{norm} that was the same to within uncertainty, and a BIC that was only 1.7 higher compared to the original best fit, therefore this equally valid fit was chosen since it was more compatible with the other samples. Furthermore, the SLD profile above the SiO_2 was the same as in the original fit, within uncertainty. Models with an additional layer to allow for either a surface layer or a more complicated interface or gradient structure were tried. However, they did not result in an improved fit for physically possible profiles. These fits indicate that the large gradient is preferred between the SAM and the main Nafion layer.

These SiO_2 parameters were then used to fit the unannealed hydrated neutral Amine SAM data (which was taken before the sample was annealed). Models 12 and 11 with either 3 or 4 layers above the SiO_2 (see figure S2, left and right respectively) allowed for an extra interface layer, a gradient layer, or a surface layer. These showed a similar profiles except that the 4 layer model had and extra 5.2nm thick layer below the main layer that had a slightly lower SLD than the main layer, which was shallower than the gradient seen in the dry case. The water rich SAM layer was nearly identical in both fits, in thickness 2.11 [2.05,2.18] nm vs. 2.13 [2.06,2.18] nm and SLD 0.70 [0.61,0.79] 10^{-4} nm^{-2} vs 0.72 [0.62,0.79] 10^{-4} nm^{-2}. Also the fitted Nafion volume fraction of the main layer was similar for the 3 layer and 4 layer fits, 0.8195 [0.815, 0.825] vs 0.8377 [0.833,0.843] respectively.

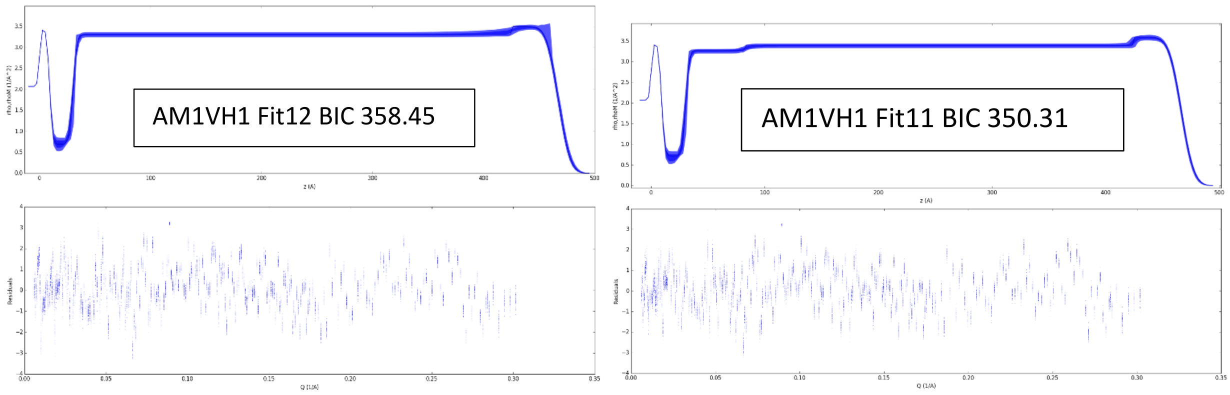

Figure S2 Fits to the hydrated unannealed neutral A-SAM. Left, the 3 layer model and right, the four layer model

The surface layer in the 4 layer model was 4.2nm thick and had a slightly higher SLD than in the 3 layer fit. See Figure S2 for both. Since the 4L fit has a BIC of 350.31 which was lower than the 3L by 8.1, it is considered a slightly favored solution, *but the presence of the shallow interfacial layer is not definitively required*. However, the best fit without the surface layer has a χ^2_{norm} =1.17261, and BIC=383.1, indicating that the surface layer is required.

In the case of the neutral amine sample in the hydrated state *after* the annealing at 120° C, the data was also fit with models that have the SiO_2 determined from the dry fit followed by either 3 layers or 4 layers. In both cases the layer thickness and SLD ranges allowed them to be either extra interfacial or gradient layers or a surface layer. The 4 layer fit, fit 7, was selected because it had a much better χ^2_{norm} (1.242 vs. 1.547) and BIC (474.35 vs 556.78) than the 3 layer fit, fit 5. The profiles however were similar, with the added layer of the 4layer model being a thin, 0.84nm layer with SLD of 2.2x10^{-4} nm^{-2} similar to the SAM layer of the 3layer fit. However, the next layer after that had a lower SLD of 0.294x10^{-4} nm^{-2} and thickness of 1.049nm. The combined thickness of the first 2 layers above the SiO_2 in the 4 layer fit was 1.89nm, similar to the thickness of the SAM layer in the 3 layer model of 1.99nm. So the extra layer can be interpreted as modifying the profile of the HG to be non-uniform, with a lower SLD region further from the SiO_2. The best fit without the surface layer has a χ^2_{norm} =1.690, and BIC=601.8, indicating that the surface layer is also strongly required.

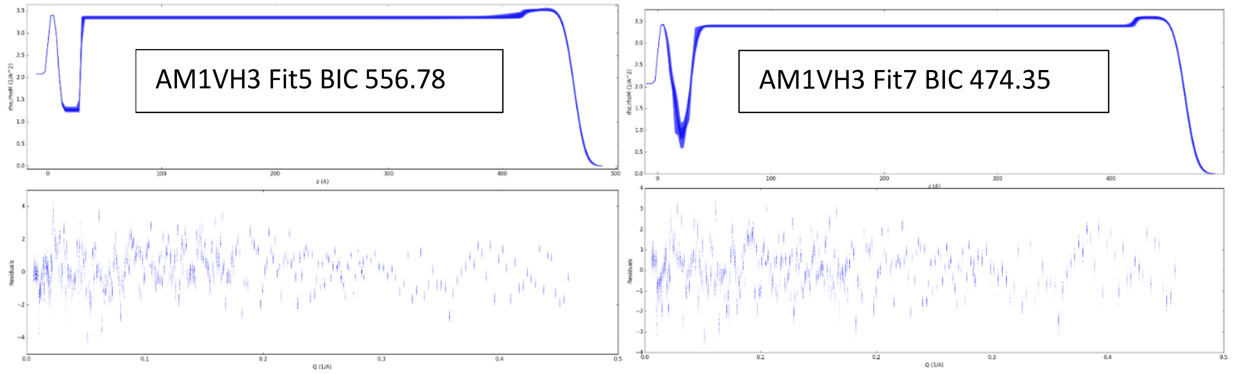

Figure S3 Fits to the hydrated annealed neutral amine sample.

S1.6 Fitting the positive A-SAM data

Because the positive amine SAM was a separate sample, the SiO_2 layer parameters were determined from fits of the data taken in the humidified condition, the only data set collected. Because of the large number of interfacial layers, both Damped Oscillator and Independent Layer models were tried. The Damped Oscillator model (fit 14) established the number of significant interfacial lamellae to be 5 including the SAM layer and provided a baseline structure. The Independent Layer models with this number of layers were fit to the data and resulted in a similar profile, with a lower χ^2_{norm}, implying a better fit (Fit 19), but BIC was 494.3 which was slightly higher than the DO model (BIC=484.6) due to more fitting parameters, but still only larger by 9.7. However, due to the better fit χ^2_{norm} and more general approach (which can negate the BIC argument to the extent that the models cannot both

produce exactly the same SLD profile), and only insignificantly worse BIC, the independent layer fit was considered the better representation of the sample. Specifically, the DO model had one more Nafion rich layer but the SLD uncertainty band in this region overlapped that of the non-interfacial layer. Since the IL model had a lower χ^2_{norm}, the ability to independently determine the SLDs of the remaining layers has a larger effect on minimizing the residuals than the presence of the extra non-significant Nafion rich layer of the DO. This indicates that the IL best fit has the right number of interfacial lamellae. In both of these models the first water poor layer had a very high SLD. The center of its uncertainty band is consistent with the SLD of Fluorocarbons without any sulfonic acid side chains or water, not seen in other samples. This is however possible, and phase segregation of the FC backbone from the sulfonic acid side chain has been observed before[3] although in that study the sample did have some water in the layer.

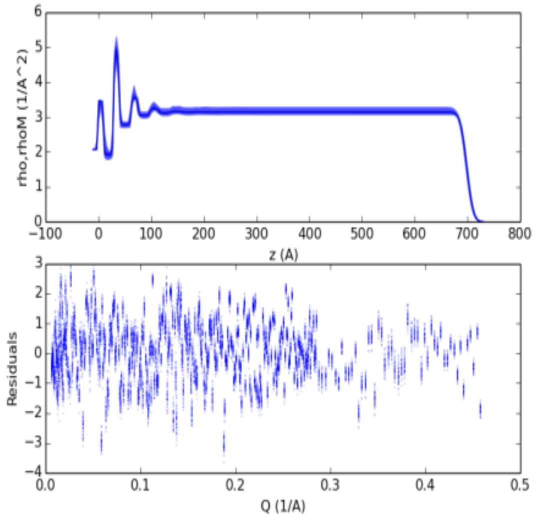

a) Fit 14 Damped Oscillator Model χ^2_{norm} =0.993 BIC 484.6

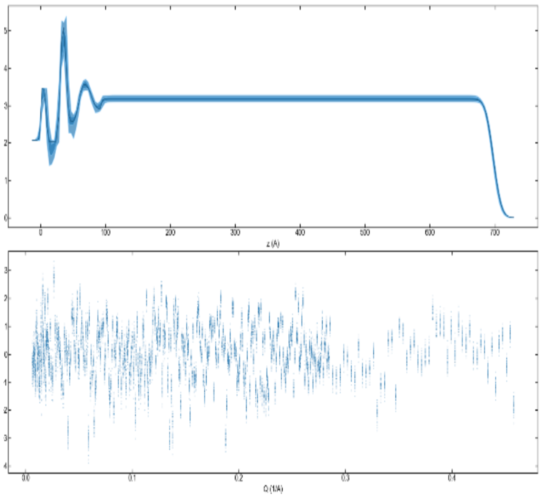

b) Best fit, Fit 19 χ^2_{norm} = 0.965(73) BIC=494.3

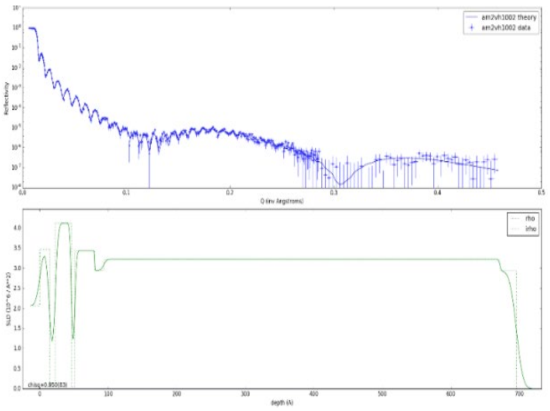

c) Fit with Surface layer, fit21 χ^2_{norm} = 0.950(83) BIC= 503.90 (Because the uncertainty bands are large and obscure the best fit profile, this plot is shown instead

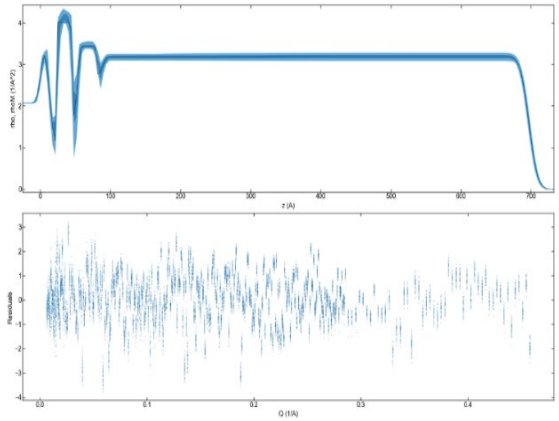

d) Fit 16 , another fit with Lower SLD Lamellaeχ^2_{norm} = 0.974(83) BIC=512.6

Figure S4 Fits to the positive A-SAM

Adding a layer that could fit to a gradient or interfacial or surface layer, Fit 21 (Figure S4c) produced a fit with the Nafion volume fraction of the surface layer at 0.741 [0.70, 0.87] insignificantly smaller than (i.e. within the 68% confidence interval of) the non-interfacial layer, 0.802 [0.75, 0.92]. Furthermore, the slight rounding of the SLD profile in fit 21 can also be interpreted as a modification of the roughness profile than an actual surface layer. Because the SiO_2 layer had a broad interface with the Si, a sharp interface with the SAM, and lower SLD than the bulk, unlike the rest of the samples, this layer model was rejected. Furthermore, this model had a BIC of 503.9 that was 18 higher than the best fit. Another model Fit 16 (Figure S4d) with a similar SiO_2 and SAM layer but without the surface layer had a BIC of 512.6 that is also 18.3 higher than the best fit, and was also rejected due to the non-physical and inconsistent SiO_2. However, the final interpretation of the best fit profile can be tempered by these nearly good fits, i.e. a lower SLD surface layer, better interpreted as an odd roughness profile, and the slightly different profile of the DO model, and a 1st water poor layer that had SLD closer to that of Bulk Nafion than Fluorocarbons.

References:

(1) Dura, J. A.; Murthi, V. S.; Hartman, M.; Satija, S. K.; Majkrzak, C. F. Multilamellar Interface Structures in Nafion. *Macromolecules* **2009**, *42* (13), 4769–4774. https://doi.org/10.1021/ma802823j.

(2) DeCaluwe, S. C.; Baker, A. M.; Bhargava, P.; Fischer, J. E.; Dura, J. A. Structure-Property Relationships at Nafion Thin-Film Interfaces: Thickness Effects on Hydration and Anisotropic Ion Transport. *Nano Energy* **2018**, *46*, 91–100. https://doi.org/10.1016/j.nanoen.2018.01.008.

(3) DeCaluwe, S. C.; Kienzle, P. A.; Bhargava, P.; Baker, A. M.; Dura, J. A. Phase Segregation of Sulfonate Groups in Nafion Interface Lamellae, Quantified via Neutron Reflectometry Fitting Techniques for Multi-Layered Structures. *Soft Matter* **2014**, *10* (31), 5763–5776. https://doi.org/10.1039/C4SM00850B.

(4) Kirby, B. J.; Kienzle, P. A.; Maranville, B. B.; Berk, N. F.; Krycka, J.; Heinrich, F.; Majkrzak, C. F. Phase-Sensitive Specular Neutron Reflectometry for Imaging the Nanometer Scale Composition Depth Profile of Thin-Film Materials. *Curr. Opin. Colloid Interface Sci.* **2012**, *17* (1), 44–53. https://doi.org/10.1016/j.cocis.2011.11.001.

(5) Kienzle, P. A.; Maranville, B. B., Refl1d Github/Refl1D, https://Github.Com/Reflectometry/Refl1d/Releases (accessed 2025-07-28).

(6) Kienzle, P. A.; Maranville, B. B. Github/Bumps, https://github.com/bumps/bumps (accessed 2025-07-28).

(7) Dura, J. A.; Rus, E. D.; Kienzle, P. A.; Maranville, B. B. Nanolayer Analysis by Neutron Reflectometry. In *NANOLAYER RESEARCH: METHODOLOGY AND TECHNOLOGY FOR GREEN CHEMISTRY*; Elsevier, 2017; pp 155--202.

(8) Nevot, L.; Croce, P. *Rev Phys Appl* **1980**, *15*, 761–779.

(9) Kass, R. E.; Raftery, A. E. Bayes Factors. *J. Am. Stat. Assoc.* **1995**, *90* (430), 773–795. https://doi.org/10.1080/01621459.1995.10476572.

(10) Bass, M.; Berman, A.; Singh, A.; Konovalov, O.; Freger, V. Surface Structure of Nafion in Vapor and Liquid. *J. Phys. Chem. B* **2010**, *114* (11), 3784–3790. https://doi.org/10.1021/jp9113128.

(11) Dowd, R. P.; Day, C. S.; Van Nguyen, T. Engineering the Ionic Polymer Phase Surface Properties of a PEM Fuel Cell Catalyst Layer. *J. Electrochem. Soc.* **2017**, *164* (2), F138–F146. https://doi.org/10.1149/2.1081702jes.
